# Linalool Prevents Cisplatin Induced Muscle Atrophy by Regulating IGF-1/Akt/FoxO Pathway

**DOI:** 10.3389/fphar.2020.598166

**Published:** 2020-11-30

**Authors:** Hong Zhang, Mengyi Chi, Linlin Chen, Xipeng Sun, Lili Wan, Quanjun Yang, Cheng Guo

**Affiliations:** ^1^ Department of Pharmacy, Shanghai Jiao Tong University Affiliated Shanghai Sixth People’s Hospital, Shanghai, China; ^2^ School of Medicine, Shanghai Jiao Tong University, Shanghai, China; ^3^ School of Pharmacy, Shanghai University of Traditional Chinese Medicine, Shanghai, China

**Keywords:** natural products, forkhead box O, muscle atrophy, cachexia, cisplatin, linalool

## Abstract

Skeletal muscle atrophy is an important feature of cancer cachexia, which can be induced by chemotherapy, and affects the survival and quality of life of cancer patients seriously. No specific drugs for cancer cachexia have been applied in clinical practice. This study explored the therapeutic effect of linalool (LIN) on cisplatin (DDP) induced skeletal muscle atrophy. *In vivo*, LIN can improve skeletal muscle weight loss, anorexia, muscle strength decline and other cachexia symptoms caused by cisplatin treatment in a Lewis lung cancer tumor bearing mouse model, and cause no adverse effects on the anti-tumour effect. LIN treatment decreased the expression of muscle RING-finger protein-1 (MuRF1) and Atrogin1(MAFbx) in muscle, and the activation of insulin-like growth factor-1 (IGF-1)/protein kinase B (Akt)/forkhead box O (FoxO) pathway was observed. *In vitro*, LIN alleviated DDP induced C2C12 myotube atrophy, and IGF-1 receptor inhibitor Picropodophyllin (PIC), which had no adverse effect on C2C12 myotube cells, could reverse the protective effect of LIN. These results indicate that LIN down-regulates the expression of Atrogin1 and MuRF1 through the IGF-1/Akt/FoxO pathway, alleviating DDP-induced muscle atrophy and improving cachexia symptoms. LIN has the potential to be developed as a drug against cancer cachexia.

## Introduction

Linalool (LIN, C_10_H_18_O, molecular weight: 154.25 g mol^−1^) is a monoterpene with multiple pharmacological effects and is derived from many fruits, aromatic plants and Chinese herbal medicines, such as *Citrus reticulata peel*, *Linderae Radix* and *Amomum aurantiacum*. LIN has an inhibitory effect on colon cancer, glioma and liver cancer cells (Iwasaki et al., 2016; Cheng et al., 2017; Rodenak-Kladniew et al., 2018) and can enhance the curative effect of anthracyclines in the treatment of breast cancer (Ravizza et al., 2008). LIN also has anti-inflammatory effects (Huo et al., 2013; Kim et al., 2019), inhibiting inflammation of the respiratory tract, and can reduce inflammation-related pain (Berliocchi et al., 2009). By inhibiting oxidative stress, LIN can protect nerves and reduce cerebral ischemic injury (Park et al., 2016; Sabogal-Guáqueta et al., 2019). In terms of psychiatric efficacy, LIN also has effects against anxiety and depression (Pereira et al., 2018).

Chemotherapy is widely used in the treatment of cancer (Frei, 1985), but many chemotherapy drugs induce cachexia while suppressing tumors. Clinical studies have shown that patients with lung cancer will experience weight loss, BMI decrease, and sarcopenia after chemotherapy (Nattenmuller et al., 2017). In patients with advanced epithelial ovarian cancer, the skeletal muscle index decreased significantly after platinum-based chemotherapy started (Huang et al., 2020). Muscle loss in cancer patients during chemotherapy is associated with poor prognosis (Daly et al., 2018; Griffin et al., 2019) and poor chemotherapy treatment effects (Sasaki et al., 2019). Relieving muscle loss and cachexia during chemotherapy is of great significance for patient survival.

It is acknowledged that activation of protein degradation and defection of skeletal muscle regeneration induce muscle atrophy. The activation of the ubiquitin ligase family members Atrogin1 (MAFBx/FBX32) and muscle RING finger protein 1 (MuRF1) induce protein degradation and plays an important role in muscle wasting (Bodine and Baehr, 2014; Sukari et al., 2016). Simultaneously, the expression of myogenic factors, such as MyHC, MyoG, and MyoD, is downregulated, while that of negative myogenic factors, such as myostatin, is upregulated (Cohen et al., 2015; Bossola et al., 2016). Interventions targeting proteins related to myogenesis and degradation can effectively improve skeletal muscle atrophy; for example, strategies regulating myostatin, growth hormone (GH)/insulin-like growth factor-1 (IGF-1) and phosphatidylinositol 3-kinase (PI3K)/protein kinase B (Akt)-mediated anabolic pathways can be used to relieve skeletal muscle damage (Dutt et al., 2015). Forkhead box O family member proteins (FoxOs) are highly conserved transcription factors with important roles in cellular homeostasis. Activation of FoxO leads to Atrogin1 induction, and IGF-1 treatment or Akt overexpression can inhibit FoxO and Atrogin1 expression (Sandri et al., 2004).

As a commonly used drug for the treatment of lung cancer, cisplatin (DDP) can lead to skeletal muscle atrophy and simulate the cachexia caused by chemotherapy. It has been reported that DDP can decrease the phosphorylation of Akt and FoxO3α, which leads to the blockade of the upregulation of MuRF1 and Atrogin1, and activate protein degradation pathway (Sakai et al., 2014). While, regulating IGF-1/Akt/FoxO pathway can alleviate DDP induced muscle atrophy (Sirago et al., 2017). The mechanism of skeletal muscle damage caused by DDP has many similarities with cancer cachexia (Moreira-Pais et al., 2018). As a model, DDP induced cachexia in tumour-bearing animals can be used to screen anti-cancer cachexia drugs. On this basis, tumour-bearing animals can also be used to assess the effect of drugs on tumor growth.

At present, there are no reports about LIN alleviating cancer cachexia, especially chemotherapy-induced cachexia. The aim of this study was to investigate the effect of LIN on DDP-induced cachexia and the mechanism by which LIN alleviates skeletal muscle atrophy. The primary objective was to explore the potential of LIN in the treatment of skeletal muscle atrophy in DDP-induced cachexia.

## Materials and Methods

### Chemicals

Linalool (LIN; L812404, purity: >98%, Macklin, Shanghai, China), picropodophyllin (PIC; HY-15494, purity: >99%, MedChemExpress, NJ, United States) were prepared in dimethylsulfoxide (DMSO; D8371, purity: >99%, Solarbio, Beijing, China); cisplatin (DDP; HY-17394, purity: >99%, MedChemExpress, NJ, United States) was prepared in phosphate buffered saline (PBS, P1020, pH: 7.2-7.4, Solarbio, Beijing, China) for experiments *in vitro* and normal saline (0B92A2, China Otsuka Pharmaceutical Co. Ltd, Tianjin, China) for experiments *in vivo*. All the solution above was stored at –20°C.

### Animals

Four-week-old C57BL/6 male mice were purchased from SLAC Laboratory Animal Co. Ltd (Shanghai, China) and allowed 1 week of adaptation before the study began. Mice were maintained under 23 ± 1°C with 12 h light/dark cycles with free access to water and regular chow diet. All animal experiments were performed in compliance with the ethical requirements of the Laboratory Animal Research Center, Shanghai Jiao Tong University Affiliated Shanghai Sixth People’s Hospital. The experimental protocol was approved by Animal Welfare Ethics Committee of Shanghai Sixth People’s Hospital (NO: DWLL2020-0550).

### DDP-Induced Muscle Atrophy and LIN Treatment Regimen

**FIGURE 1:**
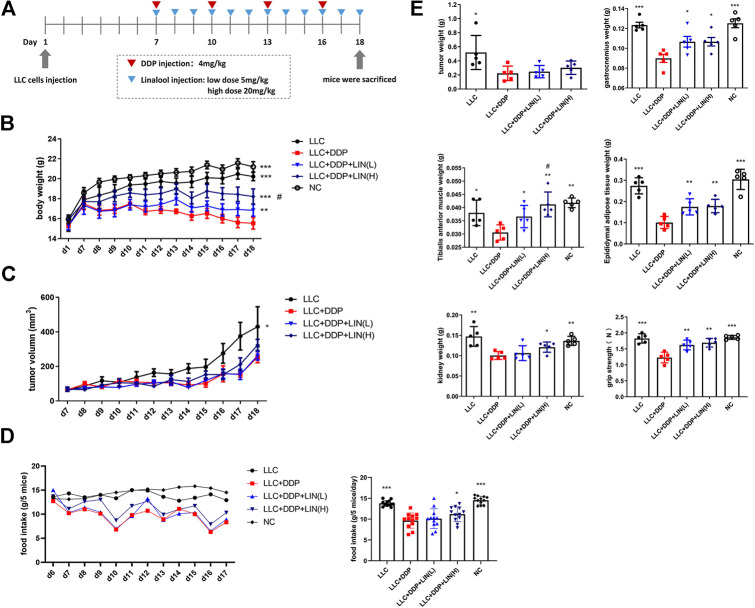
LIN alleviated the decrease of body weight, food intake, muscle, adipose tissue, kidney weight and forelimb grip of tumor bearing mice after DDP treatment without affecting the anti-tumour effect of DDP **(A)** 5-week-old C57BL/6 mice were treated with DDP (4 mg kg^−1^·3 days^−1^, i.p.) and given high and low dose of LIN (5, 20 mg kg^−1^·d^−1^, i. p.) at the same time lasted for 12 days **(B)** Body weight was measured on day 1 and day 7–18 (n = 60); **p < 0.01, ***p < 0.001 vs. LLC + DDP group, #*p* < 0.05 vs. Group LLC + DDP + LIN(L). **(C)** The tumor size was measured daily on day 7-18. **(D)** Food intake of each group of mice was measured daily form one day before dosing to the end (left panel). Data are shown as mean ± S.E.M. (n = 12); *p < 0.05, ***p < 0.001 vs. LLC + DDP group. **(E)** Mice were sacrificed, the tumor, gastrocnemius muscle, tibial anterior muscle, epididymal fat, and kidney were collected and weighed, and the forelimb grip strength was measured before the mice were sacrificed. Data are shown as mean ± S.E.M. (n = 5); *p < 0.05, **p < 0.01, ***p < 0.001 vs. Group LLC + DDP, #*p* < 0.05 vs. Group LLC + DDP + LIN(L).

Twenty five C57BL/6 male mice (5-week-old, mean body weight: 20 g) were randomly divided into five groups: normal control (NC), LLC, LLC + DDP (DDP, 4 mg kg^−1^·3 days^−1^, i. p.), LLC + DDP + LIN (L) (DDP, 4 mg kg^−1^·3 days^−1^, i. p.; LIN, 5 mg kg^−1^ d^−1^, i. p.), LLC + DDP + LIN (H) (DDP, 4 mg kg^−1^·3 days^−1^, i. p.; LIN, 20  mg kg^−1^ d^−1^, i. p.). On day 1, 1 × 10^6^ mouse Lewis Lung cancer (LLC) cells were injected subcutaneously into the right flank of mice except Group NC. The stock solution of LIN (20, 80 mg mL^−1^) was diluted in corn oil (1:10) and DDP (0.8 mg mL^−1^) prepared in normal saline were administered on day 7-18 (Figure 1A). Group NC, LLC were administered with the same volume of solvent (DDP solvent: normal saline, LIN solvent: 10% DMSO in corn oil). On day 18, the grip strength of the mice was tested. The mice were then sacrificed for tissue collection. Tumors, Gastrocnemius (GA), Tibialis anterior (TA) muscle, epididymal adipose, kidneys were collected and weighed. During the course of the experiment, the body weight, tumor volume and food intake of the mice were checked daily between 12:00-14:00.

### Cell Culture and Treatment

Murine C2C12 myoblasts (GNM26) and LLC cells were kindly provided by Stem Cell Bank, Chinese Academy of Sciences. The C2C12 myoblasts were cultured in Dulbecco’s modified Eagle’s medium (DMEM; 10-013-CV, Corning Life Sciences, NY, United States) supplemented with 10% fetal bovine serum (10,141, Gibco, MA, United States), and penicillin-streptomycin (100 units ml^−1^) (P1400, Solarbio, Beijing, China). Cells were cultured in 5% CO2 at 37°C. To differentiate C2C12 myoblasts into myotubes, C2C12 myoblasts were seeded at the density of 5×10^5^ cells per well in 6-well plate. When the cells grew to a density of 80%–90%, the medium was replaced with differentiation medium (DMEM supplemented with 2% horse serum (16050122, Gibco, MA, United States) and penicillin-streptomycin (100 units ml^−1^) (P1400, Solarbio, Beijing, China)) for 4-6 days. The differentiation medium was replaced every 48 h. After differentiation the C2C12 myotubes were treated with 40 μM DDP and/or 25 μM LIN for 24 h, then harvested for further tests. Another group of C2C12 myoblasts were seeded in 6-well plate at the same density. When the differentiation procedure started, the C2C12 myoblasts were treated with 2.5 μM DDP and/or 25 μM LIN for 0, 48, 96 h, then harvested for further tests.

### CCK8 Assay

C2C12 myoblasts (1 × 10^4^ cells/well) in 96-well plates were differentiated into myotubes (The differentiation procedure was same as described above). The C2C12 myotubes and another group of C2C12 myoblasts without differentiation were treated with different concentrations of LIN on triplicate wells for 24, 48, 72 h. Cell proliferation was determined using Cell Counting Kit-8 (CCK-8, Dojindo Molecular Technologies, Kumamoto, Japan) by adding 100 µL CCK-8 reagent (10 μg mL^−1^) to each well for 1 h at 37°C, and measured the optical density (OD) at 450 nm by a plate reader (BioTek, VT, United States).

### Grip Strength Measurement

On Day 18, mice were subjected to measure grip strength. Limb grip strength was measured using a grip strength meter (SH-20, NSCING, Nanjing, China). mice were allowed to rest on a metal mesh that they could grip by two forelimbs. The tail of each mouse was pulled parallel to the metal mesh three times. The maximum force of the grab process was recorded, and the maximum force value was used to reflect muscle force.

### RNA Isolation, Reverse Transcription, and Quantitative Real-Time Polymerase Chain Reaction

Total RNA was isolated from the GA muscle using RNAsio Plus (9109, TAKARA, Kyoto, Japan) according to the manufacturer’s protocol. Complementary DNA (cDNA) was synthesized from 2000 ng total RNA with the HiScript II Q Select RT SuperMix (R223-01, Vazyme, Nanjing, China). qRT-PCR was performed using a reaction mixture containing SYBRTM Green master mix (Q711-02-AA, Vazyme, Nanjing, China). Results were calculated using the 2-△△CT relative quantification method normalized to the 18S gene. The sequences of the primer pairs are shown in Supplementary Table S1.

### Western Blot Analysis

The C2C12 myotubes and gastrocnemius muscle were lyzed using RIPA buffer (89,900, Thermo Fisher Scientific, MA, United States) containing a protease inhibitor mixture (P0100, Solarbio, Beijing, China) and phosphatase inhibitor mixture (4906837001, Roche, Basel, Swiss) according the manufacturer’s instructions. Nucleoprotein and cytoplasm protein was extracted using nuclear protein extraction kit (R0050, Solarbio, Beijing, China). Lysates were centrifuged at 14,000 rpm for 10 min in 4°C. After evaluating the protein concentration using a bicinchoninic acid protein assay kit (P0011, Beyotime, Haimen, China), protein was denatured in 5×loading buffer (C516031, Sangon, Shanghai, China). A 30 µg protein was loaded, and separated by 10% sodium dodecyl sulfate polyacrylamide gel, subsequently transferred to nitrocellulose filter membranes (Millipore Corporation, Bedford, United States). After blocked in tris buffered saline (TBS) containing 5% BSA at room temperature for 2 h, the membranes were probed by the primary antibodies at 4°C overnight, followed by incubated with IR-Dye680-conjugated anti-mouse or anti-rabbit secondary antibody (926-68,071, LI-COR Biosciences, NE, United States) (dilution: 1:5000) for 1 h at room temperature. The information of primary antibodies (dilution: 1:1000) is as follows: anti-MYH (B-5) (sc-376157, Santa Cruz biotechnology, CA, United States), anti-MuRF1 (ab172479, Abcam, MA, United States), anti-Atrogin1/MAFbx (ab168372, Abcam, MA, United States), anti-*p*-Akt (Tyr308) (2965, Cell Signaling Technology, MA, United States), anti-*p*-Akt (Ser473) (4060, Cell Signaling Technology, MA, United States), anti-Akt (9272, Cell Signaling Technology, MA, United States), anti-*p*-FoxO3α (Ser253) (9466, Cell Signaling Technology, MA, United States), anti- FoxO3α (12,829, Cell Signaling Technology, MA, United States), anti-Tubulin (2128, Cell Signaling Technology, MA, United States), anti-MyoG (ab1835, Abcam, MA, United States), anti-MyoD (ab126726, Abcam, MA, United States), anti-Myostatin/GDF8 (ab203076, Abcam, MA, United States), anti-Lamin B1 (12,987, Proteintech, CHI, United States), anti-IGF1R (9750, Cell Signaling Technology, MA, United States), anti-IRS1 (3407, Cell Signaling Technology, MA, United States). Finally, membranes were visualized by Odyssey infrared imaging system (LI-COR Biosciences, NE, United States), anti-Ubiquitin (ab179434, Abcam, MA, United States), anti-GAPDH (5174, Cell Signaling Technology, MA, United States). Tubulin, GAPDH or Lamin B1 were used as internal control.

### Histology

GA muscles were fixed in 4% PBS-buffered paraformaldehyde and embedded in paraffin. These paraffin blocks were cut into 4 μm thick sections and stained with hematoxylin and eosin (H&E) kit (G1121, Solarbio, Beijing, China). The H&E-stained sections were used for the cross-sectional area (CSA) analyses. Muscle sections were captured and evaluated using Cellsens software. Fiber CSA of gastrocnemius muscles was acquired from assessment of 100 fibers per mouse.

### Immunoblotting

The C2C12 myotubes were fixed with 4% PBS-buffered paraformaldehyde for 15 min at room temperature, then permeabilized with 0.5% Triton X-100 for 20 min. After blocked in 5% bovine serum albumin at room temperature for 1 h, the C2C12 myotubes were incubated with anti-MYH primary antibody (sc-376157, Santa Cruz biotechnology, CA, United States) overnight at 4°C, followed by incubated with mouse Alexa Fluor^®^ 488 conjugate (4408, Cell Signaling Technology, MA, United States) antibody for 1 h, then 10 μg/ml 4, 6-diamidino-2-phenylindole (DAPI; D9542,Sigma-Aldrich, St Louis, United States) for 5 min. PBS was used to wash 3 times between each operation, and each time for 5 min. At last photographed using the Cellsens software (Olympus Corporation, Tokyo, Japan).

### Statistical Analysis

Statistical analysis was performed by GraphPad Prism v8.3 software (GraphPad Software Inc., San Diego, CA, United States). Data were expressed as mean ± standard error of the means (S.E.M.) or standard deviation (S.D.). For comparison of two sets of measurements, *t* test was performed. For comparison of three or more sets of measurements, two-way ANOVA followed by post hoc comparison with Tukey’s test were performed. *p* < 0.05 was considered statistically significant.

## Results

### Linalool Alleviated DDP-Induced Cachexia in Lewis Lung Cancer Tumor Bearing Mice

In order to explore the effect of LIN on LLC tumor bearing mice treated with DDP, 5-week-old C57BL/6 mice were treated with DDP (4 mg kg^−1^·3 days^−1^, i. p.) and divided into groups treated with LIN (5, 20  mg kg^−1^ d^−1^, i. p.) for 12 days (Figure 1A). LIN could alleviate the weight loss induced by DDP in a dose-dependent manner (Figure 1B). The tumor size in the group receiving DDP treatment was smaller than that in the Group LLC significantly, and there was no significant difference in the tumor size between the groups receiving LIN treatment and Group LLC + DDP (Figure 1C). After counting the daily food intake of each group of mice, it was found that high-dose LIN could increase the food intake of mice after DDP treatment (Figure 1D). LIN alleviated the weight loss of gastrocnemius (GA) muscle and tibialis anterior (TA) muscle after DDP treatment, enhanced the grip strength of forelimbs, and prevented weight loss of epididymal adipose and kidney (Figure 1E).

### Linalool Relieved Muscle Fiber Atrophy Induced by DDP

**FIGURE 2:**
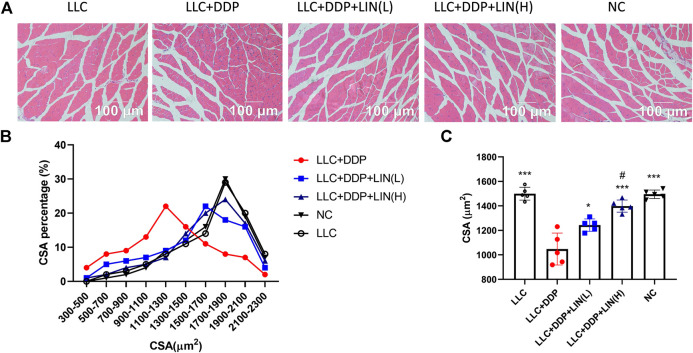
LIN increases the CSA of muscle fibers in mice after DDP treatment. The sections from the GA muscle were stained with H&E and observed under a microscope **(A)** a group of representative pictures are shown. **(B)** Muscle fiber size distribution. **(C)** The CSA was measured using Cellsens software and the mean CSA of GA muscle from each mouse has been represented (n = 5). ***p < 0.001 *vs.* Group LLC + DDP, #*p* < 0.05 vs. Group LLC + DDP + LIN(L).

It has been reported that DDP can induce skeletal muscle fiber atrophy (Conte et al., 2017; Chen et al., 2019). We evaluated the effect of LIN on the muscle fiber after DDP treatment *in vivo*. The GA muscle was stained with H&E and the cross-sectional area (CSA) of muscle fibers was analyzed. As shown in Figure 2A, C, the CSA of GA muscle fibers in Group LLC + DDP + LIN (L) and Group LLC + DDP + LIN (H) was significantly larger than that in Group LLC + DDP. In Group LLC + DDP, the highest proportion of CSA interval was 900-1500 μm^2^, about 51%, and the mean CSA value was 1082 μm^2^. In Group LLC + DDP + LIN (L), the highest CSA interval was 1300-1900 μm^2^, about 52%, and the mean CSA value was 1316 μm^2^. In Group LLC + DDP + LIN (H), the highest CSA interval was 1500-2100 μm^2^, about 61%, and the mean CSA was 1441 μm^2^ (Figures 2B,C). LIN alleviated the reduction of CSA of GA muscle fibers in a dose-dependent manner.

### Effect of Linalool on the microRNA and Protein Expression of Myosin Heavy Chains and Muscle Specific E3 Ubiquitin-Protein Ligases in Gastrocnemius Muscle

**FIGURE 3:**
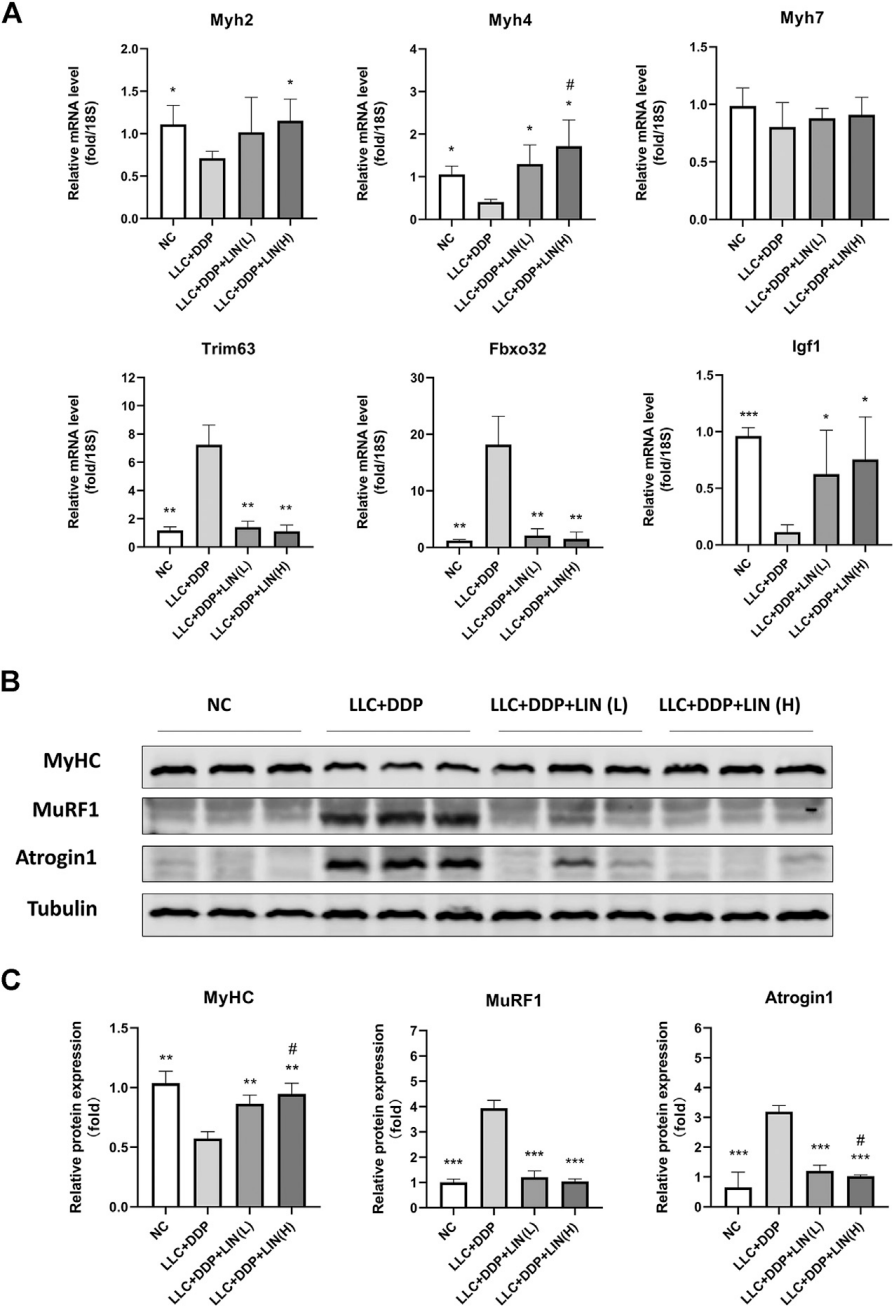
LIN up regulated the expression of myogenesis related factors in GA muscle and down regulated the expression of muscle degradation related factors **(A)** The mRNA expression of Myh2, 4, 7, Trim63, Fbxo32, Igf1 in the GA muscle was assessed by qRT-PCR, 18S was used as an internal control. **(B)** Protein expression of MyHC, MuRF1, Atrogin1 was evaluated by western blotting, Tubulin was used as an internal control. **(C)** The relative expression levels of the proteins were quantified using ImageJ software and normalized to Tubulin and corrected to Group NC. Data shown as mean ± S.E.M. (n = 3); *p < 0.05, **p < 0.01, ***p < 0.001 vs. Group LLC + DDP, #*p* < 0.05 vs. Group LLC + DDP + LIN(L).

Skeletal muscle is composed of different types of muscle fiber. Different types of muscle fiber express different myosin heavy chain (MyHC), such as MyHC I (slow, oxidative), MyHC IIA (fast, oxidative) and MyHC IIB (very fast, glycolytic) encoded by genes Myh7, Myh2 and Myh4 respectively (Schiaffino, 2018). In cancer cachexia, the expression of MyHC of fast-twitch muscle types is down-regulated considerably more than slow-twitch muscle types (Ciciliot et al., 2013). Skeletal muscle degradation is often accompanied by the up-regulation of muscle specific E3 ubiquitin-protein ligase MuRF1 and Atrogin1 (Johns et al., 2013). In this study, LIN increased the mRNA expression of Myh2 (MyHC 2A), Myh4 (MyHC 2B), and Igf1 in the GA muscle, and the mRNA expression of Myh4 was up-regulated in a dose-dependent manner. The mRNA expression of Trim63 (MuRF1), Fbxo32 (Atrogin1) was significantly down-regulated by LIN treatment. While there was no significant change in the mRNA expression of Myh7 (MyHC I) (Figure 3A). The protein expression of MyHC in GA muscle was up-regulated in a dose-dependent manner (Figures 3B,C). The protein expression of MuRF1, Atrogin1 and ubiquitin was down-regulated, only Atrogin1 showed a dose-dependent manner (Figures 3B,C and Supplementary Figures S2A,B). IGF-1/PI3K/Akt pathway is one of the main pathways involved in skeletal muscle hypertrophy (Johns et al., 2013). The intervention of LIN up-regulated the expression of IGF-1 mRNA, suggesting that IGF-1/Akt pathway may be related to the anti-muscle atrophy effect of LIN.

### Linalool Up-Regulated the Expression of pAkt and pFoxO in Gastrocnemius Muscle

**FIGURE 4:**
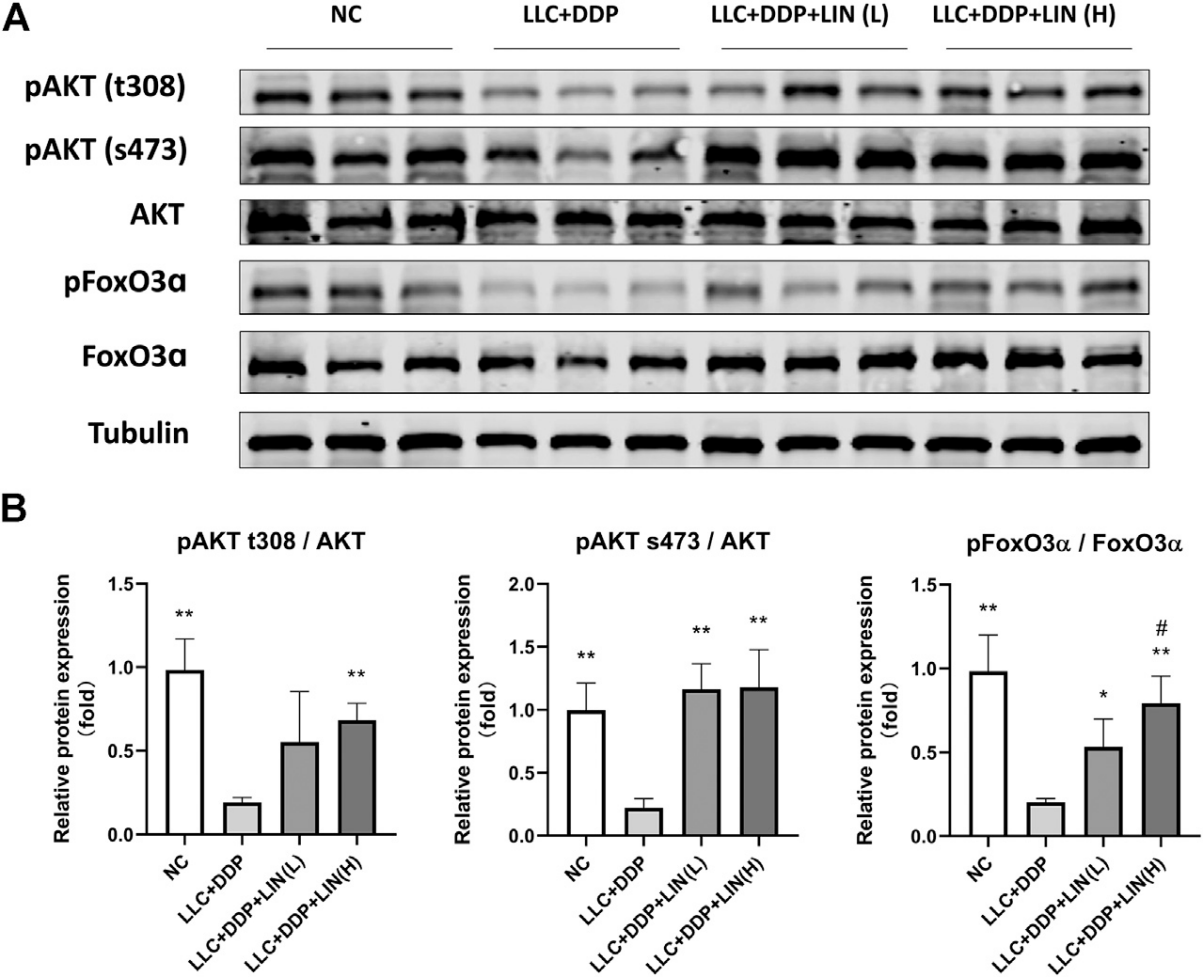
LIN activated Akt/FoxO pathway in GA muscle **(A)** Protein expression of MyHC, MuRF1, Atrogin1 was evaluated by western blotting, Tubulin was used as an internal control. **(B)** The relative expression levels of the proteins were quantified using ImageJ software and normalized to Tubulin and corrected to Group NC. Data shown as mean ± S.E.M. (n = 3); *p < 0.05, **p < 0.01 vs. Group LLC + DDP, #*p* < 0.05 vs. Group LLC + DDP + LIN(L).

Akt/FoxO pathway can regulate the expression of MuRF1 and Atrogin1, and activating Akt can inhibit FoxO from entering the nucleus to regulate the transcription of MuRF1 and Atrogin1 by phosphorylation of FoxO (Johns et al., 2013), so we detected the expression levels of pAkt and pFoxO3α. LIN activated the Akt/FoxO pathway, and the up-regulation of FoxO3α phosphorylation was in a dose-dependent manner (Figures 4A,B).

### Effect of Linalool on Differentiation and Degradation of DDP Treated C2C12 Myoblasts and Myotubes

**FIGURE 5:**
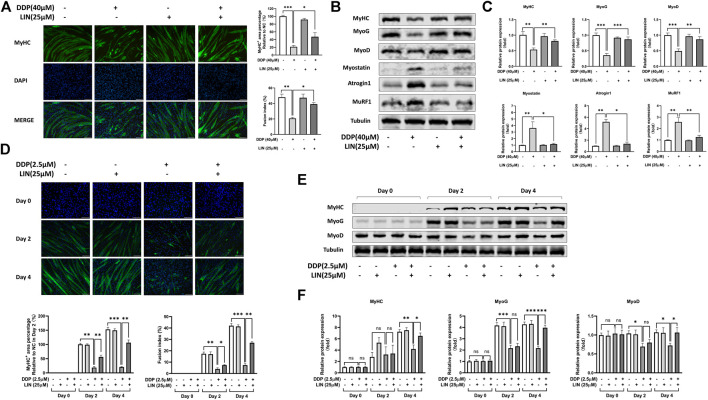
Effect of LIN on differentiation and atrophy of DDP treated C2C12 myotubes **(A)** The MyHC^+^ area and fusion index of C2C12 myotubes were measured using ImageJ software (scale bars = 100 μm). **(B)** Protein expression of MyHC, MyoG, MyoD, Myostatin, Atrogin1, MuRF1 was evaluated by western blotting, Tubulin was used as an internal control. **(C)** The relative protein expression levels of MyHC, MyoG, MyoD, Myostatin, Atrogin1, MuRF1 were quantified using ImageJ software and normalized to Tubulin and corrected to the untreated group. **(D)** The MyHC^+^ area and fusion index of C2C12 myotubes in differentiation process were measured using ImageJ software (scale bars = 100 μm). **(E)** Protein expression of MyHC, MyoG, MyoD was evaluated by western blotting, Tubulin was used as an internal control. **(F)** The relative protein expression levels of MyHC, MyoG, MyoD were quantified using ImageJ software and normalized to Tubulin and corrected to the untreated group. Data shown as mean ± S.E.M. (n = 3); *p < 0.05, **p < 0.01, ***p < 0.001.

We investigated the effects of LIN and DDP on the viability of C2C12 myoblasts and myotubes to determine the dosage *in vitro*. As shown in Supplementary Figures S1A,C, LIN has a weak cytotoxic effect on C2C12 myoblasts and myotubes, and the dosage of LIN also determined by referring to previous studies (Park et al., 2016; Cheng et al., 2017). However, DDP has a strong cytotoxic effect on cell viability (Supplementary Figures S1B,D). The 24 h dose of DDP on C2C12 myotubes was determined according to the previous studies (Chen et al., 2015; Chen et al., 2019), while the dose in the progress of C2C12 myoblasts differentiating into myotubes was 2.5 μM, which has a lower cytotoxic effect. As shown in Figure 5A, LIN prevented the differentiated C2C12 myotubes atrophy induced by DDP. Myogenin (MyoG), myogenic differentiation 1 (MyoD), and Myostatin (MSTN) play an important role in C2C12 myotube differentiation. MyoG, MyoD are positive related factors, while MSTN can inhibit myotube growth and differentiation (Rodriguez et al., 2014). The intervention of LIN increased the expression of MyHC, MyoG, and MyoD, and down-regulated the expression of MSTN, MuRF1, Atrogin1 and ubiquitin in C2C12 myotubes (Figures 5B,C and Supplementary Figures S2C,D). LIN can also reduce the inhibition of DDP on C2C12 myoblast differentiation (Figure 5D), and up-regulate the protein expression of MyHC, MyoG and MyoD in DDP treated C2C12 myotubes (Figures 5E,F).

### Linalool Prevented Forkhead Box O From Entering Nucleus in DDP Treated C2C12 Myotubes, and the Therapeutic Effect of Linalool Could Be Blocked by IGF1-R Inhibitor

**FIGURE 6:**
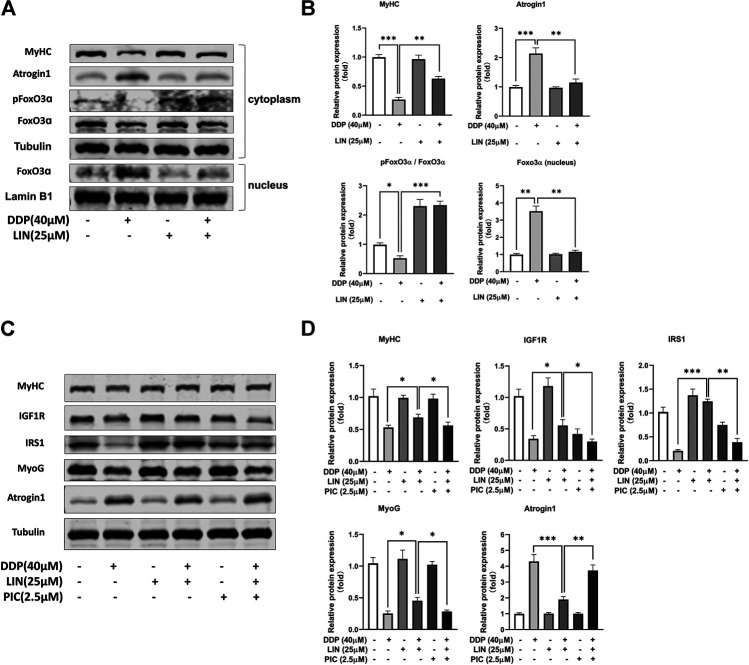
LIN prevented FoxO from entering nucleus and alleviated muscle atrophy in DDP treated C2C12 myotubes, which could be blocked by IGFR inhibitor PIC **(A)** Cytoplasm protein expression of MyHC, Atrogin1, pFoxO3α, Foxo3α was evaluated by western blotting, Tubulin was used as an internal control. Nucleus protein expression of Foxo3α was evaluated by western blotting, Lamin B1 was used as an internal control. **(B)** The relative expression levels of the proteins were quantified using ImageJ software, the cytoplasm protein relative expression levels were normalized to Tubulin and corrected to the untreated group, the nucleus protein relative expression levels were normalized to Lamin B1 and corrected to the untreated group. **(C)** The protein expression of MyHC, IGF1R, IRS1, MyoG, Atrogin1 was evaluated by western blotting, Tubulin was used as an internal control. **(D)** The relative expression levels of the proteins were quantified using ImageJ software and normalized to Tubulin and corrected to the untreated group. Data shown as mean ± S.E.M. (n = 3); *p < 0.05, **p < 0.01, ***p < 0.001.

In C2C12 myotubes treated with DDP, LIN can phosphorylate FoxO, inhibit FoxO from entering the nucleus, and reduce the expression of FoxO in the nucleus, thereby inhibiting the expression of Atrogin1 and up-regulating the expression of MyHC (Figures 6A,B). IGF1/Akt/FoxO pathway can be blocked by IGF1R inhibitor picropodophyllin (PIC) (George et al., 2019). In this study, PIC did not up-regulate the expression of Atrogin1 and down-regulate the expression of MyHC. The results showed that PIC can block the therapeutic effect of LIN (Figures 6C,D), indicating that LIN relies on the IGF1/Akt/FoxO pathway to exert anti-muscle atrophy effect.

## Discussion

Platinum drugs are nonspecific anti-tumour drugs that can destroy DNA function and inhibit cell mitosis. DDP, a typical platinum drug, is widely used in the treatment of lung cancer, head and neck cancer, ovarian cancer and bladder cancer (Dasari and Tchounwou, 2014). However, resistance to DDP can occur, and DDP has many adverse side effects, such as kidney injury, gastrointestinal disease, muscle atrophy and progressive cachexia symptoms (Moreira-Pais et al., 2018). In this study, due to the short growth cycle of the tumors, the tumor load in Lewis lung carcinoma (LLC)-bearing mice was light, and no cachexia symptoms, such as weight loss, anorexia and fatigue, occurred. Treatment with DDP provided benefits in terms of slowing tumor growth, but these benefits were also accompanied by the occurrence of cachexia (Conte et al., 2020). Reducing the side effects of chemotherapy drugs is of great significance for successful cancer treatment.

Some studies have shown that the skeletal muscle atrophy caused by DDP can be improved by drugs. Growth hormone secretagogues (GHSs) can alleviate DDP-induced changes in skeletal muscle calcium homeostasis (Conte et al., 2017). The natural product *phytocannabinoid cannabigerol* (CBG) can improve anorexia symptoms and skeletal muscle atrophy in rats after DDP treatment (Brierley et al., 2019). There are also some Chinese herbal medicines that can prevent the skeletal muscle atrophy caused by DDP. The standardized extract of Panax ginseng can alleviate the symptoms of cachexia induced by DDP (Lobina et al., 2014). *Scutellaria baicalensis* (SB) Georgi extract can not only alleviate the skeletal muscle atrophy caused by DDP but also reduce kidney damage (Huang et al., 2019). Modified Sijunzi decoction can reduce DDP-induced skeletal muscle mitochondrial dysfunction (Chen et al., 2019). As such, obtaining medicinal components for preventing and treating cachexia from Chinese herbal medicines or natural products is feasible. LIN can be derived from a variety of Chinese herbal medicines, spices, and fruits. It is widely distributed in nature and easy to obtain. Research on the effects of LIN against cachexia is helpful for exploring the beneficial effects of Chinese herbal medicines and food in cachexia.

In this study, LIN alleviated the weight, skeletal muscle, and adipose tissue loss of a cachexia mouse model generated by DDP treatment, maintained muscle function, and reversed the decrease in food intake. In the analysis of gastrocnemius mRNA, DDP may cause the atrophy of fast-twitch glycolytic fibers. The diameter of fast-twitch glycolytic fibers was larger than that of slow-twitch oxidative fibers; as such, the gastrocnemius muscle slices showed a significant reduction in the cross-sectional area of muscle fibers. Fast-twitch glycolytic fibers are more vulnerable than slow-twitch oxidative fibers to a variety of atrophic conditions related to Forkhead box O (FoxO) family signaling, autophagy inhibition, transforming growth factor beta family signaling, and nuclear factor-κB (Wang and Pessin, 2013). However, detection of mRNA expression alone is not enough to directly explain the reason for the change of skeletal muscle CSA. It is not possible to rule out the reduction of skeletal muscle CSA caused by the conversion of fast-twitch fibers to slow-twitch. In the future study, we can further identify the changes of muscle fiber types by specific staining combined with immunohistochemistry. In the analysis of gastrocnemius mRNA, it was found that the expression of Igf-1 was affected by LIN treatment, and IGF-1 expression was closely related to FoxO expression, which is related to skeletal muscle atrophy. FoxO proteins are a subclass of the FoxO superfamily of transcription factors (TFs). They can interact with the promoters of a variety of target genes and can regulate a series of biological activities that are vital to the maintenance of cell homeostasis, such as cell proliferation, energy production, and resistance to oxidative stress (Zhang et al., 2011; Tia et al., 2018; Link, 2019). Studies have shown that IGF-1 can activate Akt to phosphorylate FoxO proteins to inhibit their transcriptional function, which benefits cell survival, growth and proliferation and can inhibit muscle atrophy in skeletal muscle (Zhang et al., 2011; Wimmer et al., 2014). Skeletal muscle IGF-1 overexpression activates the AKT/FoxO signaling pathway, inhibits Atrogin1 expression, and reduces the skeletal muscle atrophy induced by angiotensin II (ANG II) (Yoshida et al., 2010). In addition, this study adopted a model of cachexia induced by DDP. There have been reports that DDP regulates FoxO proteins and FoxO upstream pathways to produce pharmacological effects. For example, DDP inhibits the phosphorylation of FoxO3α and induces the accumulation of FoxO3α in the nucleus by inhibiting the PI3K/Akt pathway, thus increasing the expression of the FoxO3α-dependent apoptotic protein BIM, inhibiting the proliferation of lung cancer cells and inducing apoptosis (Liu et al., 2014). However, the inhibition of the PI3K/Akt pathway by DDP also leads to skeletal muscle atrophy (Sirago et al., 2017). The effect of LIN on skeletal muscle atrophy may be related to the regulation of FoxO proteins and their upstream pathways. Therefore, we detected the phosphorylation levels of Akt and FoxO3α in gastrocnemius muscle and found that LIN promoted the phosphorylation of Akt and FoxO3α and activated the Akt/FoxO pathway.

**FIGURE 7:**
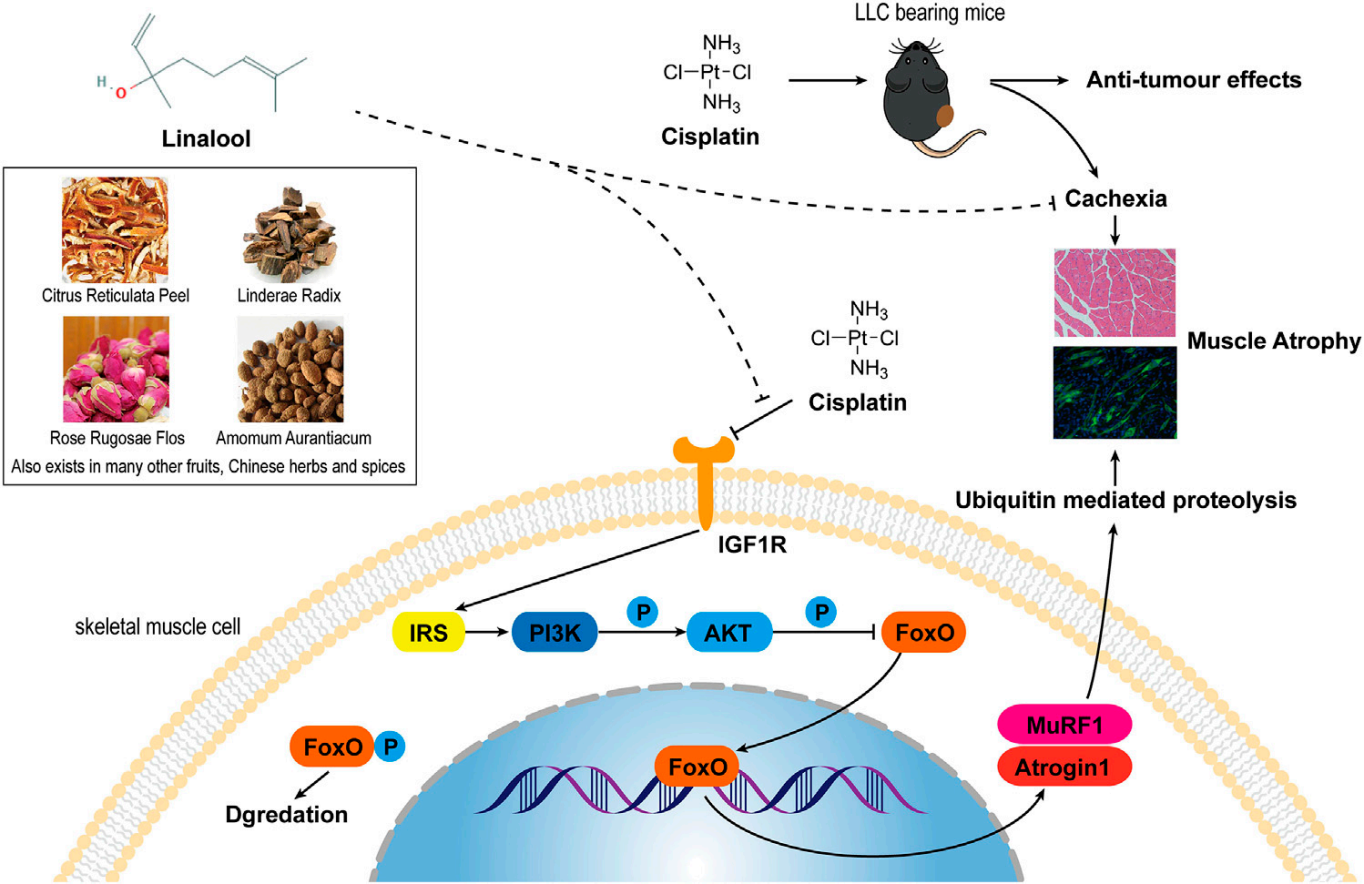
A summary of the effect and mechanism of the anti-DDP-induced muscle atrophy of LIN. LIN comes from a variety of Chinese herbal medicines, fruits and spices, and is widely distributed in nature. LIN can improve the cachexia symptoms of DDP-treated tumour-bearing mice and reduce muscle atrophy without affecting the anti-tumor efficacy of DDP. The mechanism is that LIN can down-regulate the expression of Atrogin1 and MuRF1 through the IGF-1/Akt/FoxO pathway to alleviate muscle atrophy.

To further clarify the mechanism of action of LIN, we used an *in vitro* skeletal muscle atrophy model, C2C12 myotube cells treated with DDP. Studies have reported that DDP can induce atrophy of differentiated C2C12 myotubes and can also inhibit the differentiation of C2C12 myoblasts into myotubes (Wu et al., 2019). Since LIN is still in the preclinical research stage and there are no reports containing pharmacokinetic data, it was not possible to determine the effective dose of LIN in skeletal muscle tissue. In this study, the *in vitro* experimental doses of LIN used were mainly derived from the commonly used doses in other pharmacological studies of LIN, and the cytotoxic effect of various doses of LIN on C2C12 myoblasts and myotubes were verified through CCK8 assay. Ultimately, a dose that did not have serious effects on cell survival was selected for the experiments, that is, 25 μM. The results showed that LIN could prevent the myotube atrophy and poor differentiation induced by DDP, upregulate the expression of MyoG, MyoD and MyHC, and inhibit the expression of myostatin, Atrogin1, MuRF1 and ubiquitin. LIN inhibited the expression of FoxO3α in the nucleus and promoted the phosphorylation of FoxO3α in the cytoplasm. After blocking the IGF-1/Akt/FoxO signaling pathway with the IGF1R inhibitor PIC, which initially had no negative effect on C2C12 myotubes, the therapeutic effect of LIN was reversed, indicating that the skeletal muscle atrophy-alleviating effect of LIN depended on the IGF-1/Akt/FoxO signaling pathway. A summary of the mechanism of anti-DDP-induced muscle atrophy of LIN is shown in Figure 7.

Importantly, when LIN activated the IGF-1/Akt/FoxO signaling pathway to inhibit skeletal muscle degradation, it showed no inhibition of the anti-tumour effect of DDP. This may be because LIN itself has anti-tumour abilities and does not promote tumor growth. However, a synergistic inhibitory effect between LIN and DDP on tumor growth in tumour-bearing mice was not observed in this study. LIN had a protective effect on skeletal muscle without affecting the efficacy of DDP, which suggests that LIN has potential utility in the treatment of cancer cachexia related to chemotherapy.

There is still much work to be done before LIN enters the clinic. For example, in experiments *in vitro*, we also observed that LIN inhibits the expression of myostatin. The myostatin-Smad2/3 pathway is a negative regulator of protein synthesis. Inhibition of this pathway will also have benefits in terms of relieving skeletal muscle atrophy (Schiaffino et al., 2013; Gallot et al., 2014; Biglari et al., 2020). Whether LIN has an effect on Smad proteins remains to be clarified. In addition, systemic inflammation is also an important driving factor in the occurrence and development of cachexia. In previous reports, LIN was shown to have anti-inflammatory effects. The regulation of inflammation may also be one of the mechanisms by which LIN exerts skeletal muscle atrophy-alleviating effects. After each injection of cisplatin, the food intake of mice decreased obviously, while high-dose LIN could alleviate this effect. The increase of food intake may promote the maintenance of body weight and muscle weight of mice. In the future study, the daily food supply of mice can be restricted to balance the food intake of different groups, so as to exclude the influence of food intake on body weight and muscle. Last but not least, LIN is volatile, and optimization of the dosage should be performed before LIN is applied in the clinic to enable collection of accurate pharmacokinetic data in phase I clinical trials.

## Conclusion

LIN can down-regulate the expression of Atrogin1 and MuRF1 through the IGF-1/Akt/FoxO pathway to alleviate DDP-induced muscle atrophy. At the same time, LIN will not affect the anti-tumour effect of DDP, and can also improve cachexia symptoms such as anorexia, fatigue, weight loss caused by DDP treatment.

## Data Availability Statement

The raw data supporting the conclusions of this article will be made available by the authors, without undue reservation.

## Ethics Statement

The animal study was reviewed and approved by Animal Welfare Ethics Committee of Shanghai Sixth People’s Hospital (NO: DWLL2020-0550).

## Author Contributions

HZ, QY, and CG designed the study. HZ, MC, LC, XS, and LW performed the experiments and analyzed data. HZ and MC wrote the original draft. QY and CG contributed to the revision of manuscript. All authors approved the final manuscript.

## Funding

This work was supported by the National Natural Science Foundation of China (Nos. 81873042, 81872494 and 81803633).

## Conflict of Interest

The authors declare that the research was conducted in the absence of any commercial or financial relationships that could be construed as a potential conflict of interest.
