# Supplementary material for: Linalool Prevents Cisplatin Induced Muscle Atrophy by Regulating IGF-1/Akt/FoxO Pathway
Source: Front Pharmacol. 2020 Nov 30;11:598166. doi: 10.3389/fphar.2020.598166 (PMC7774296; doi:10.3389/fphar.2020.598166)
Supplement: Supplementary file 1 [file datasheet1.docx]

Supplementary Material

**TABLE S1** Primers used for qRT-PCR analysis

| **Target** | **Primer Sequence (5’ to 3’)** |
| --- | --- |
| Myh2 F | GCGACAGACACCTCCTTCAAGAAC |
| Myh2 R | GTCCAGCCAGCCAGTGATGTTG |
| Myh4 F | TGATGCAGGCTGAGATCGAGGAG |
| Myh4 R | TTGGTGTTGATGAGGCTGGTGTTC |
| Myh7 F | GCAAGACGGTGACTGTGAAGGAG |
| Myh7 R | GGTTGACGGTGACGCAGAAGAG |
| Fbxo32 F | GTCGGCAAGTCTGTGCTGGTG |
| Fbxo32 R | AGGCAGGTCGGTGATCGTGAG |
| Mstn F | CGATGAGCACTCCACGGAATCC |
| Mstn R | ACACTCTCCTGAGCAGTAATTGGC |
| Igf1 F | GCTCTGCTTGCTCACCTTCACC |
| Igf1 R | ACACTCATCCACAATGCCTGTCTG |
| MuRF1 F | AGTGTCCATGTCTGGAGGTCGTTT |
| MuRF1 R | ACTGGAGCACTCCTGCTTGTAGAT |
| 18S F | GTAACCCGTTGAACCCCATT |
| 18S R | CCATCCAATCGGTAGTAGCG |

FIGURE S1


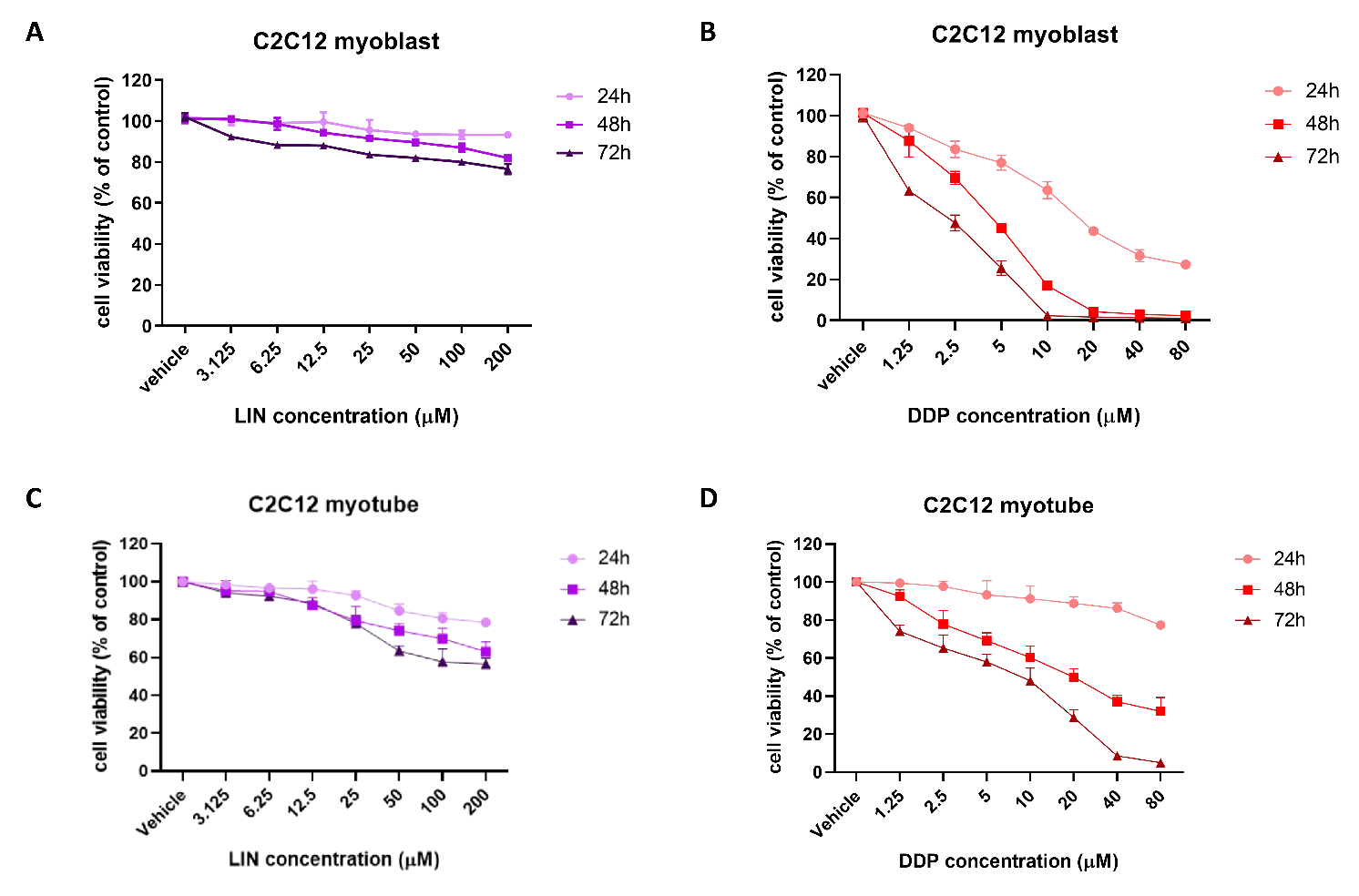


**FIGURE S1** Effects of LIN and DDP on the viability of C2C12 myoblasts and myotubes **(A)** Effects of different concentrations of LIN on the viability of C2C12 myoblasts at 24 h, 48 h and 72 h. **(B)** Effects of DDP of different concentrations on the viability of C2C12 myoblasts at 24 h, 48 h and 72 h. **(C)** Effects of different concentrations of LIN on the viability of C2C12 myotubes at 24 h, 48 h and 72 h. **(D)** Effects of DDP of different concentrations on the viability of C2C12 myotubes at 24 h, 48 h and 72 h.

FIGURE S2
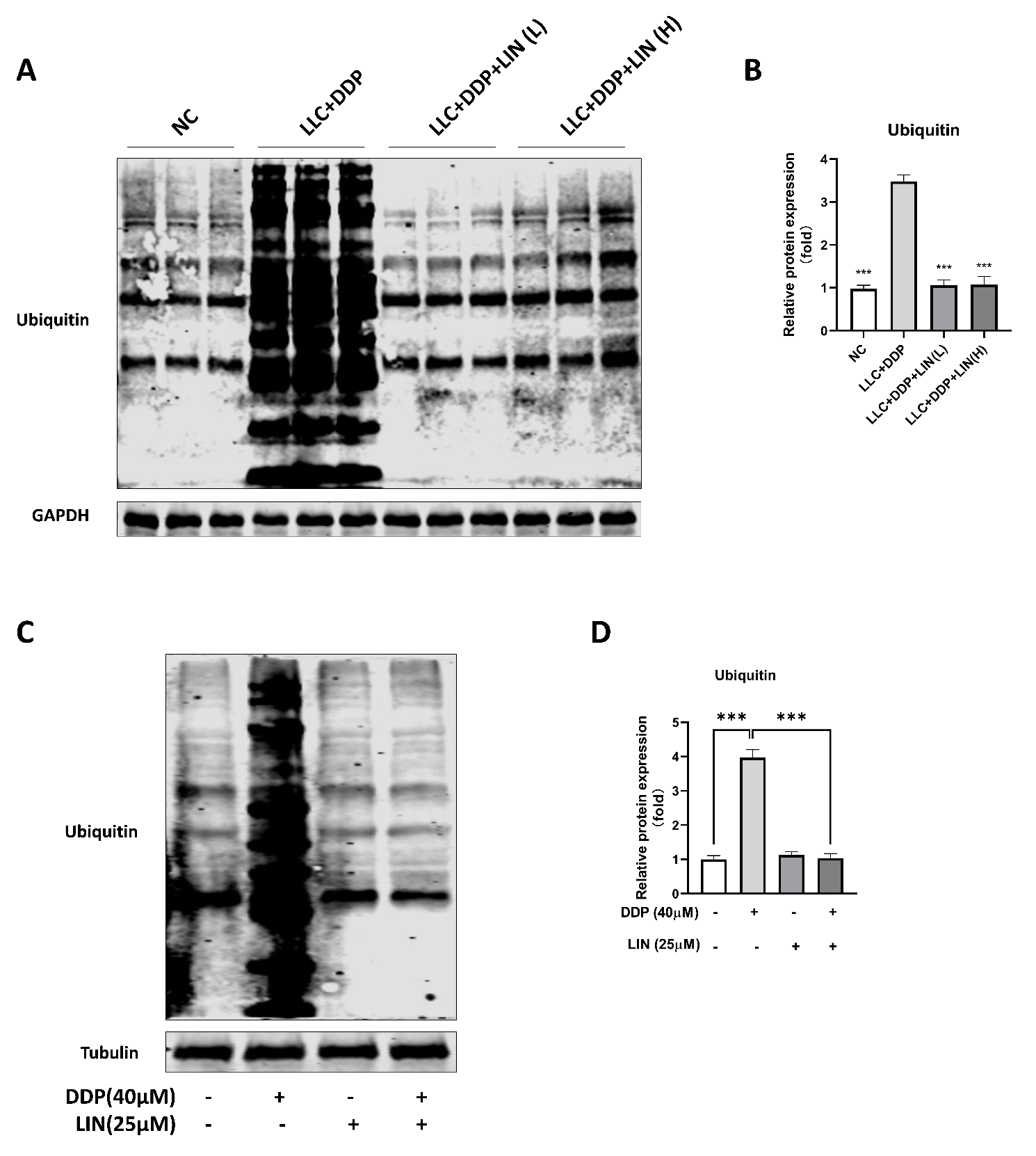


**FIGURE S2** LIN inhibits ubiquitin protein expression in mouse gastrocnemius muscle and C2C12 myotubes after DDP treatment **(A)** Protein expression of ubiquitin in mouse gastrocnemius muscle was evaluated by western blotting, GAPDH was used as an internal control. **(B)** The relative expression levels of the protein were quantified using ImageJ software and normalized to GAPDH and corrected to Group NC. **(C)** Protein expression of ubiquitin in C2C12 myotubes was evaluated by western blotting, Tubulin was used as an internal control. **(D)** The relative expression levels of the protein were quantified using ImageJ software and normalized to Tubulin and corrected to the untreated group. Data shown as mean ± S.E.M. (n=3); *** *P* < 0.001.
